# User Preferences in a Carrageenan-Based Vaginal Drug Delivery System

**DOI:** 10.1371/journal.pone.0054975

**Published:** 2013-01-24

**Authors:** Bangde Li, Toral Zaveri, Gregory R. Ziegler, John E. Hayes

**Affiliations:** 1 Sensory Evaluation Center, College of Agricultural Sciences, The Pennsylvania State University, University Park, Pennsylvania, United States of America; 2 Department of Food Science, College of Agricultural Sciences, The Pennsylvania State University, University Park, Pennsylvania, United States of America; Albert Einstein College of Medicine, United States of America

## Abstract

Topical microbicides are a promising solution to address the global threat of HIV and other sexually transmitted infections. To be successful, a microbicide not only needs to be biologically functional but also highly acceptable to users. User acceptability of microbicides can be incorporated early in the product formulation and design process. Previous qualitative research revealed women had strong preferences regarding product shape, while preferences related to size and firmness were less clear. Here, we explored the effect of size and firmness on the acceptability of semisolid ovoid microbicide prototypes intended for vaginal use. Sexually active women (n = 74) were randomized to one of two conditions: with and without applicator. Nine different prototypes were evaluated; they were formulated to low, medium and high firmness using mixtures of kappa and iota carrageenan and potassium chloride. Three sizes were produced at each firmness level. Women manipulated all nine prototypes, rating them for perceived effectiveness, imagined ease-of-insertion and willingness-to-try on visual analog scales. The influence of size and firmness on these three outcome measures were assessed using ANOVA and response surface models. Results indicated size and firmness both influenced the outcome measures, but firmess was more influential than size. Also, the specific effects of size and firmness depended strongly on presence or absence of an applicator. Generally, women in the without applicator condition wanted a larger, firmer product. Collectively, these data suggest efforts to rationally design of microbicides for enhanced user acceptability must consider factors like size and firmness. Also, the decision to include or forego an applicator should be addressed early in the design process, as it strongly influences other design decisions.

## Introduction

HIV and other sexually transmitted infections (STIs) are global threats to public health. Although new incidences of HIV infection have stabilized and AIDS-related mortality has begun to decline worldwide as of 2010, HIV will remain a major problem for years to come: about 34 million people were living with HIV worldwide in 2010, among which 3.4 million were less than 15 years old. Women accounted for 50% of the infected population. Estimates suggest 2.7 million people were newly infected in 2010, and approximately 1.8 million people around the world died of AIDS-related health problems in 2010 [1]. Thus, development of effective prevention tools remains a high priority for public health.

Transmission via sexual activity is a primary route for new HIV infections [2]. This pattern is not restricted to traditional high-risk groups, like men who have sex with men; rather, transmission via sex has also resulted in high prevalence among heterosexual women. In urban areas of Guinea, the incidence of HIV infection among women is six times greater than men; in Benin’s largest city, Cotonou, HIV infection rates are 5 times higher in women than in men [3]. HIV infections among American women have also been increasing [4]. Providing women an effective means to prevent sexually acquired HIV infections is a priority in the global fight against AIDS.

Currently, effective HIV prevention methods designed for and controlled by women are quite limited [5]. Condoms are an effective means for preventing HIV and STIs if they are applied correctly and consistently during sex [6]–[9]. However, condom use is suboptimal in a variety of contexts due to cultural and social restrictions. In some African and Asian countries, women may lack the ability or power to negotiate condom use with their partners [10], [11]. In China, 60% of commercial sex workers were unable to negotiate condom use during sex [12]. This inability to negotiate condom use increases a woman’s risk for HIV infection via unprotected sex. Therefore, alternatives to condoms, especially those that can be controlled and initiated by women, have a strong potential public health benefit. Topical microbicides have been proposed and pursued as a means to address these challenges [13]. A microbicide is an agent containing functional components that can block HIV transmission when it is applied to the vagina or rectum prior to sex [7], [13], [14].

Although a commercially viable microbicide has not yet been developed, microbicides remain an active area of research: more than 40 formulations are being currently investigated as candidates for vaginal microbicides and 12 have been tested clinically [15], [16]. Microbicide candidates can be formulated as tablets, capsules, creams, suppositories, pessaries, foams, ointments, gels, films, tampons, vaginal rings, and douches [17]. From a rheological perspective, these candidates can be classified into two forms: solid and liquid. Current ‘gel’ formulations are not gels in the technical sense; but highly viscous non-Newtonian liquids with little elastic character [18]. Likewise, so-called ‘softgel’ capsules are only really soft when compared to hard gelatin capsules. Solid forms typically have the drawback of slow release, requiring a waiting period between insertion and coitus, which may be undesirable for the user. Imaging indicates that a traditional gelatin softgel requires an hour to fully dissolve and deploy API *in vivo* [19]. Furthermore, women react negatively to products that have a ‘plastic’ appearance [20]. In contrast, liquid forms may be immediately efficacious, but often have a limited period of activity and must be reapplied prior to each act of intercourse (which may not be feasible for some users). In addition, users often complain of leakage of creams and ‘gels’. This leaves a broad design space of viscoelastic materials that has not been adequately investigated.

Previous work indicates carrageenan-based microbicides are safe for vaginal use, as shown by the Carraguard trial in 165 women [21]. We are exploring carrageenan as a potential delivery system for other active pharmaceutical ingredient (APIs), and not as a standalone microbicide by itself. Unlike other potential materials for delivery systems (e.g., lipid based suppositories), carrageenan remains a gel in tropical conditions due to the elevated melting temperature. Carrageenan is a sulfated polymer derived from red seaweed that has been widely employed as a thickener and emulsifier. κ-carrageenan forms firm gels, while λ-carrageenan is used to help with binding and retaining moisture and viscosity. ι-carrageenan forms a heat-reversible gel, if calcium ions are present. Desirable properties can be designed by formulating carrageenan gels of varying composition and polymer content, and by adding appropriate ions, such as Ca^2+^ and K^+^. Notably, this feature facilitates rational design: once ideal sensory properties have been identified by users, formulations can be adjusted to simultaneously optimize user acceptability and biophysical characteristics such a osmolality and drug release [22]. Drug release studies on our delivery system are underway and will be reported elsewhere.

The eventual success of microbicides will be determined by their ability to prevent new HIV infections. To achieve this goal, candidate microbicides need to be not only biologically functional and safe, but also highly acceptable to end-users [14]. That is, a microbicide requires more than clinical efficacy to significantly impact the global HIV epidemic, as user compliance is critical for success in the field [23], [24]. User acceptability is a primary consideration for product adherence and effectiveness [23], [25]. Currently, user perception of microbicides or microbicide surrogates have been investigated using two different approaches: with product, where physical prototypes or surrogates are presented and evaluated ex vivo [26] or intravaginally [27], and without product, where hypothetical and conceptual product functionalities and features are described [9], [28]. Assessment of user acceptability can also identify key product attributes that drive consumer acceptability, and thus direct future product development efforts [23].

Here, we formulated and tested carrageenan-based microbicide prototypes for user acceptability. Initial findings from our focus group research [29] showed 79% of participants (44 of 56) preferred oval-shaped prototypes over other shapes. The present study aimed to explore effects of firmness and size on user acceptability of microbicide prototypes quantitatively. In this study, the primary response variable used to assess user acceptability of the prototypes was willingness-to-try (*willingness*). Secondary measures of user acceptability include perceived effectiveness (*effectiveness*), and imagined ease of insertion (*insertion*). All prototypes were evaluated *ex vivo* in the hand.

## Materials and Methods

### Overview

Seventy-four women were recruited via email to participate in a laboratory-based study of user acceptability. Participants were asked to evaluate 9 microbicide prototypes *in mano* (in their hands) in isolated test booths.

### Participants

Women were recruited from an existing recruitment database maintained by the Sensory Evaluation Center at Penn State. This database consists of a large number (800+) of age diverse men and women who have previously expressed an interest in routine testing of consumer products in our facility. Due to the sensitive nature of the project, a two stage opt-in procedure was used. First, women in the database were sent an email message briefly describing the test, and asked if they would be interested in participating. If they said yes, they were sent a second email with a link to a web-based screening questionnaire. Based on answers to the screening questionnaire, eligible individuals were invited to participate. Inclusion criteria included: a) female; b) between 18 and 55 years of age; c) reported having had vaginal sex with a man in the last 12 months; d) were willing to manipulate prototypes with their hands and evaluate them using a computer-guided assessment in an isolated test booth; e) hadn’t participated in focus groups or other studies on vaginal drug delivery system at Penn State in the past year.

Of the 74 participants, the majority (98%) self identified as white/Caucasian. Participant ages were broadly distributed: 22 were 18 to 29 years old, 9 were 30 to 39, and 43 were 40 to 55 years old. Most participants (67%) had completed at least a bachelor’s degree; 16% of participants had achieved master, doctoral, or professional degrees, and 16% indicated High School or a GED was the highest level of education attained. Regarding martial status, 3 (∼4%) women were divorced or separated; 19 (∼25%) women were never married; and 52 (∼70%) women were currently married. Number of vaginal deliveries varied across the sample: ∼48% (n = 36) had not had any vaginal deliveries. For women who had delivered children vaginally, 13.5% reported one vaginal delivery, 32.4% reported two, and 5.4% reported three or more.

### Sample Design and Preparation

**Figure 1:**
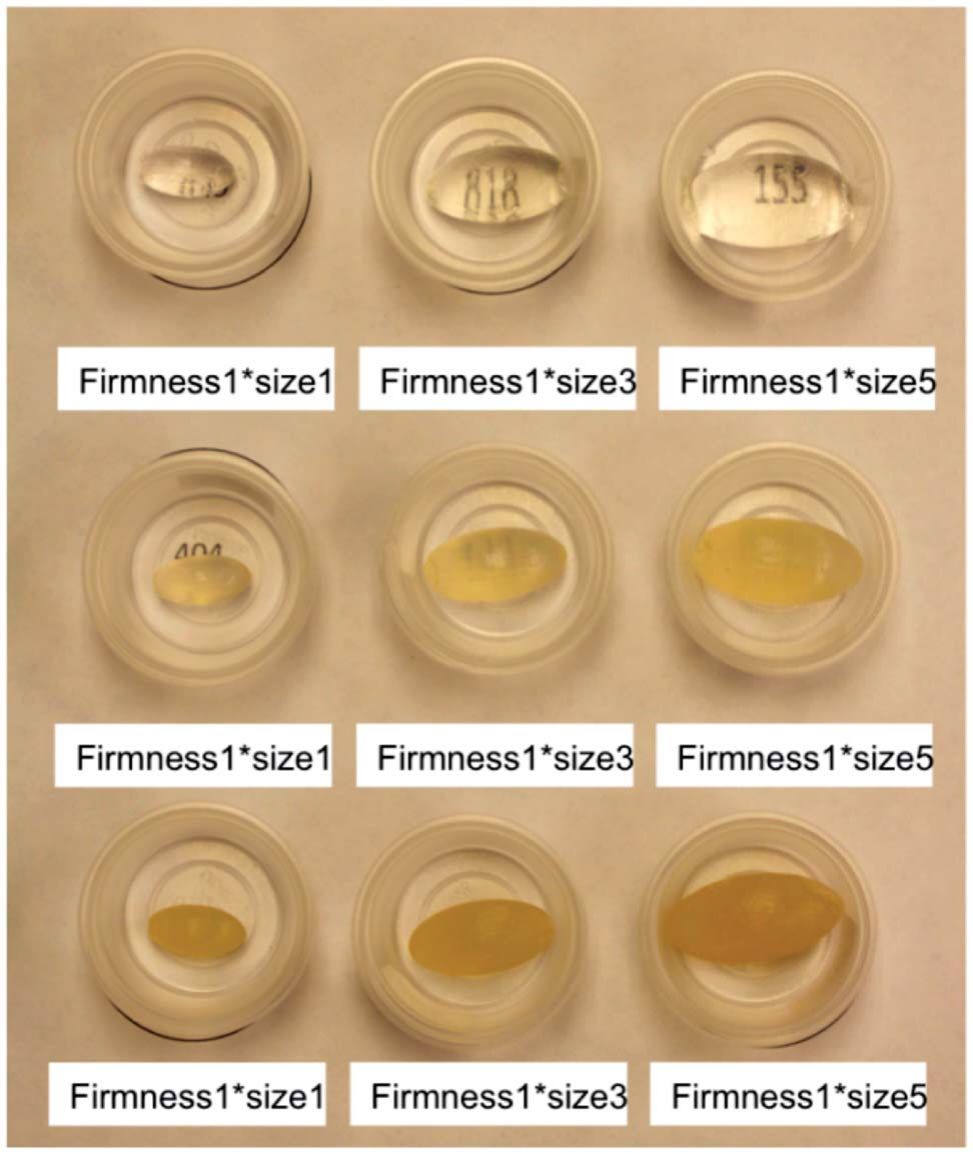
Photograph of carrageenan-based microbicide prototypes that vary systematically in size and firmness (see methods for details).

As mentioned previously, women showed a strong preference for ovoid prototypes, but were less clear regarding preferred Size and Firmness. The goal of the present study was to obtain quantitative data on nine carrageenan-based ovules that differed in Size and Firmness. A single oval shape was prepared using a full factorial design in which Size (1 g, 3 g and 5 g) and Firmness (storage modulus G’ (Pa): 1 = 250, 3 = 12500, and 5 = 125000 at 25°C, frequency at 1 rad/s, stain 1%) were varied. The length of the samples ranged from 20 mm to 34 mm, and the width ranged from 10 mm to 17 mm (Figure 1).

To prepare gels with varying Firmness levels, two types of carrageenan, kappa and iota, were mixed with potassium chloride in varying ratios (Table 1). Ingredients were mixed dry, added to deionized water, and heated in an 80°C oven. These slurries were held hot for 2–3 hours until the carrageenans were completed dissolved. During molding, the slurry was kept hot to avoid the gel setting prematurely. Hot slurries were injected into different sized molds (1 g, 3 g and 5 g) using plastic syringes. Filled molds were then moved to a refrigerator to set, which took about 15 minutes. Once the gel was set, samples were removed from the mold and put into 0.75-oz Solo transparent plastic cups (Solo Cup Company, Urbana, IL). Samples were kept at 16°C with the lids sealed tightly until the user test.

**Table 1:** Formulations of gel solution.

Formulation/Firmness	Kappa (% w/v)	Iota (% w/v )	KCl (M)	Storage modulus G′ (Pa)
1	0.1	0.9	0.06	250
2	1	1	0.1	12500
3	5	0	0.05	125000

### Product Overview and Orientation

Prior to evaluating 9 ovules in isolated test booths, participants watched a short video about the product concept in the waiting room of the Sensory Evaluation Center (Video S1). The concept video also detailed how the participant should evaluate the prototypes in her hand. Participants were instructed to: 1. Take the sample and put it into her non-dominant hand; 2. Gently stroke the sample with the index finger; 3. Put the sample between her fingers and pinch gently; 4. Finally hold the sample between her fingers and imagine she was trying to insert the sample into her vagina. The women were not provided any additional details on vaginal insertion. After watching the video, participants were provided with a paper consent form and asked to read it by project staff. After reading the consent form, women who wished to participate were given a keytag with a participant ID code and asked to enter the testing area.

### Ethics Statement

Participants provided implied informed consent after reading a paper consent form, and were reimbursed for their time. Entering the testing area from the waiting room was taken as a positive indication of consent; none of the women who watched the video and read the consent form declined to participate. Due to the potentially sensitive nature of the study, written consent was not obtained to help protect participants’ anonymity. All procedures, including the consent process, were approved by the Pennsylvania State University Institutional Review Board (protocol #36943).

### Evaluation of Samples by Participants

Data were collected using Compusense® Five software, version 5.2 (Guelph, Ontario, Canada) in individual test booths. Based on the keytag ID number, participants were randomized into one of two conditions. In one, the test computer indicated that participants should imagine the samples would be used *with* an applicator. (An example applicator was not provided, as we wanted women to react to the *idea* of an applicator rather than a specific applicator prototype that had not yet been optimized.) In the other condition, the computer told the participants they should imagine the samples would be used *without* an applicator. All other test conditions were identical across the groups. In the test booth, participants were presented 9 samples on tray in covered plastic cups labeled with 3-digit blinding codes under white light. Within a condition, sample presentation order was counterbalanced across participants using a Williams design.

After evaluating each sample, participants used a mouse to make ratings on 100 point continuous line scales (aka visual analog scales). For each sample, 3 different scales were presented on a single screen. The three scales measured perceived effectiveness (*effectiveness*), imagined ease of insertion (*insertion*), and willingness to try (*willingness*). Verbal end anchors (e.g., not at all willing, extremely willing) were provided on the scales, and these were indented at 10% and 90% of the scale to minimize end use avoidance. Demographics, such as age, education, marital status, prior usage of vaginal products, and number of vaginal deliveries, were collected at the end of the test.

### Statistical Analysis

Data were exported from Compusense® Five (Compusense Inc, Guelph, Canada) and loaded into JMP®, version 9.0.2 (SAS institute Inc, Cary NC) for further analysis. Analysis of variance (ANOVA) was employed to investigate if the factors of interest, Applicator, Size and Firmness, affected the response variables, *effectiveness*, *insertion* and *willingness*. Participants were treated as a random effect and nested in the factor of Applicator; other factors in the ANOVA model were treated as fixed effects. Higher order interactions were interpreted first, followed by less complicated interactions if the higher order interaction was not significant. Tukey HSD was used as a post-hoc testing tool to investigate factor main effect when appropriate. Response surface models were employed to assess effects of Size and Firmness on each response variable using mean ratings across participants, with separate models for each Applicator condition. These were visualized as contour plots. Pearson correlations were used to assess relationships among response variables: *effectiveness*, *insertion* and *willingness*.

## Results

Effects of Applicator, Firmness and Size of prototypes on investigated response variables, i.e., *effectiveness*, *insertion* and *willingness,* are summarized in Table 2.

**Table 2:** Effects of Applicator, Firmness and Size on *effectiveness, insertion* and *willingness*.

Main effect	*Effectiveness*		*Insertion*		*Willingness*	
	F Ratio	P-value	F Ratio	P-value	F Ratio	P-value
Applicator	1.15	0.29	9.84	0.0025	5.02	0.03
Firmness	37.17	<.0001	150.19	<.0001	63.24	<.0001
Size	10.11	<.0001	6.23	0.0021	4.90	0.01
Applicator*Firmness	11.12	<.0001	21.84	<.0001	30.70	<.0001
Applicator*Size	1.06	0.35	2.89	0.06	1.81	0.16
Firmness*Size	2.00	0.09	0.74	0.57	0.76	0.55
Applicator*Firmness*Size	0.47	0.76	1.18	0.32	0.68	0.60

Notes: terms with “*” stand for the interactions.

The model explained 59.4% of the variance in *effectiveness*. The 3-way interaction (Applicator by Firmness by Size) was not significant. Neither 2-way interaction with Size was significant, although there was a main effect of Size (Table 2). When means for *effectiveness* were compared for different sizes, it showed participants believed larger prototypes would be more effective; however, the effect was nonlinear, as perceived *effectiveness* did not differ between the largest two sizes (3 g and 5 g). The 2-way interaction between Firmness and Applicator was significant, so main effects of Firmness and Applicator were not assessed.

The model explained 65.6% of the variance in *insertion*. The 3-way interaction was not significant. The 2-way Firmness by Size interaction was not significant, and there was a main effect of Size. In contrast to *effectiveness*, there was also a marginal interaction for Applicator by Size. As with *effectiveness*, there was also a significant Applicator by Firmness interaction, so main effects were not assessed.

The model explained 64.9% of the variance in *willingness*. The 3-way interaction was not significant. Again, neither 2-way interaction with Size was significant, and there was a main effect of Size (Table 2). The 2-way interaction of Firmness by Applicator was significant, so main effects of Applicator and Firmness were not assessed.

In summary, across all three response variables, there was a simple main effect of Size. Also, there was a significant interaction between Firmness and the Applicator condition (with-applicator versus without-applicator), which indicates optimal Firmness differs whether or not the microbicide would be used with an applicator. Based on this interaction, we then modeled the effects of the design variables (Size and Firmness) separately for each applicator condition.

### Response Surface Models

To explore effects of Firmness and Size, response surface models (RSM) were created for each response variable using group means under two separate conditions: with-applicator and without-applicator.

#### Effectiveness

**Figure 2:**
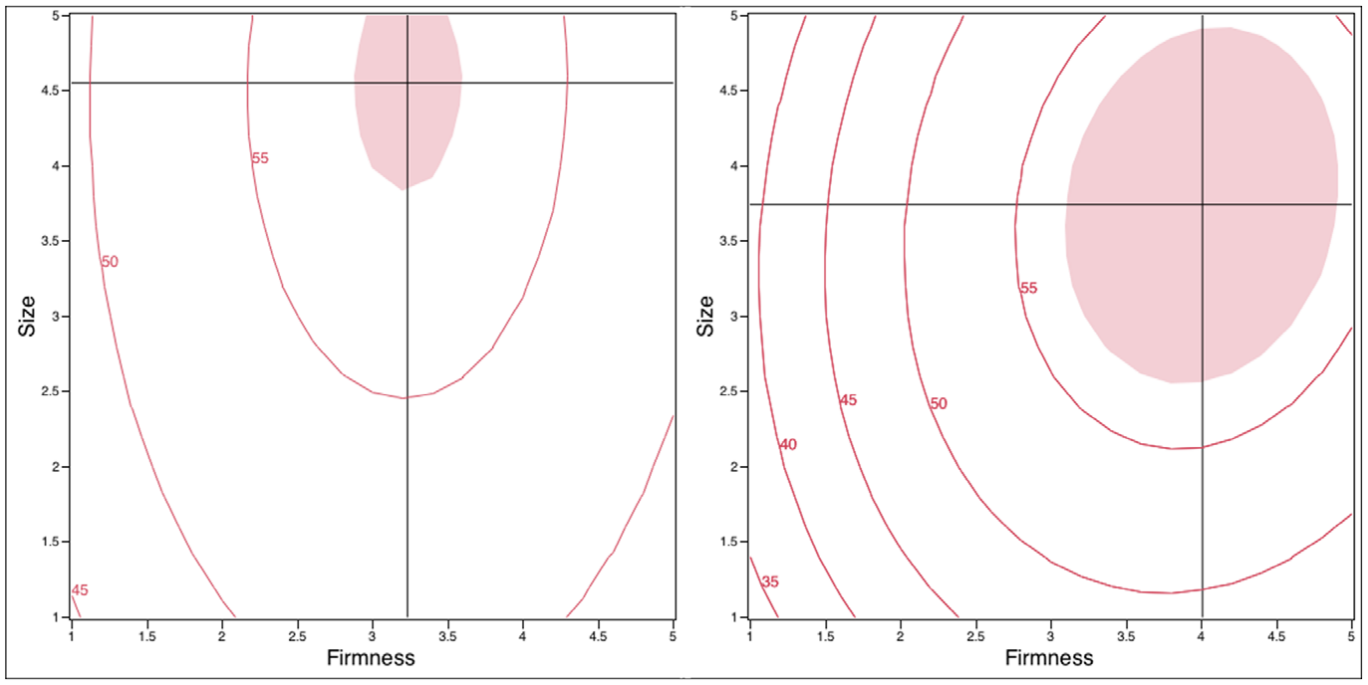
Contour plots of *effectiveness* ratings (0–100) as a function of design variables (Firmness and Size) in the with-applicator (left) and without-applicator groups (right). The cross hairs indicate the location of the maximum value and the shaded region is the same value (56.5) for both plots.

The influence of Firmness and Size on *effectiveness* were modeled as response surfaces and are displayed as contour plots in Figure 2.

For the with-applicator group (Figure 2, left), the RSM explained 89.5% of the variation in mean *effectiveness* scores. However, the overall RSM was marginal (F_5,3_ = 5.17, p = 0.10). In this model, only Firmness showed a quadratic effect (F_1,3_ = 15.29, p = 0.029). The interaction of Firmness by Size was not significant (F_1,3_ = 0.02, p = 0.899). Further, main effects, i.e., Firmness (F_1,3_ = 2.15, p = 0.239) and Size (F_1,3_ = 7.23 p = 0.074), were not significant with regard to *effectiveness* in the with-applicator group. A maximum in *effectiveness* was observed near a Firmness of 3.24 and Size of 4.55 g (Figure 2, left panel).

For the without-applicator group (Figure 2, right), 94.5% of variation in the mean *effectiveness* score was explained by the RSM (F_5,3_ = 10.31, p = 0.04). Interaction of Firmness by Size did not show a significant effect (F_1,3_ = 0.91, p = 0.411). For Firmness, the quadratic (F_1,3_ = 11.75, p = 0.042) and linear (F_1,3_ = 30.96, p = 0.012) terms were both significant. *Effectiveness* increased with increasing Firmness until Firmness reached around 4.0. Regarding Size, neither the linear (F_1,3_ = 4.09, p = 0.137) nor quadratic (F_1,3_ = 4.15, p = 0.134) terms were significant in the no-applicator group. An optimal *effectiveness* value was observed near Firmness = 4.01 and Size = 3.74 g.

When comparing users across groups (Figure 2, left vs right), women in the without-applicator group believed a slightly firmer product would be more effective. The size of an optimal microbicide for users in the without-applicator group was smaller than the one estimated in the with-applicator group.

#### Insertion

**Figure 3:**
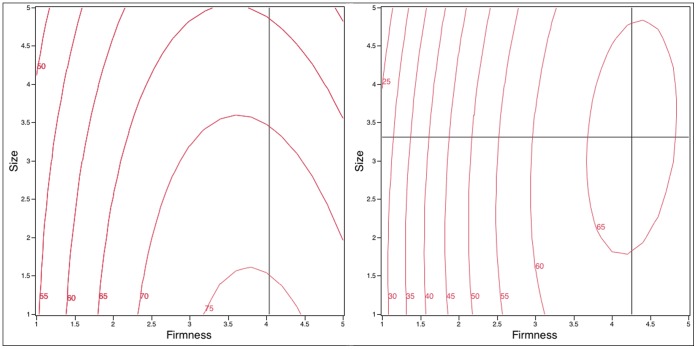
Contour plots of *insertion* ratings (0–100) as a function of design variables (Firmness and Size) in the with-Applicator (left) and without-Applicator groups (right). The cross hairs indicate the location of the maximum value; the optimal size for the with-applicator group was below 1 (outside the design space) and is not shown. A shaded region is not shown given the large differences in maximal values.

Effects of Firmness and Size on ease of *insertion* were modeled and are displayed as contour plots (Figure 3).

For the with-applicator group (Figure 3, left), the RSM explained 98.9% of the variation in mean *insertion* scores (F_5,3_ = 155.19, p = 0.0037). Interaction of Firmness by Size did not show a significant effect (F_1,3_ = 4.03, p = 0.139). For Firmness, the quadratic (F_1,3_ = 93.7, p = 0.0023) and linear (F_1,3_ = 127. 43, p = 0.0015) terms were significant. For Size, only the linear term was significant (F_1,3_ = 57.41, p = 0.0048). A maximum in *insertion* was observed near a Firmness of 4.04 (Figure 2, left panel)*;* women believed a smaller product would be easier to insert, as the optimal size fell below 1, outside the design space.

In the without-applicator group (Figure 3, right), the RSM explained 98.9% of the variation in the mean *insertion* scores and the model was significant (F_5,3_ = 57.36, p = 0.0035). There was no significant interaction for Firmness by Size (F_1,3_ = 2.64, p = 0.2029). For Firmness, the quadratic (F_1,3_ = 50.44, p = 0.0057) and linear (F_1,3_ = 229.34, p = 0.0006) terms were both significant. In contrast to the with-applicator group, Size had minimal influence for the without-applicator group, as evidenced by the non-significant linear (F_1,3_ = 0.45, p = 0.5510) and quadratic (F_1,3_ = 1.13, p = 0.3649) terms in the model; this can be seen via the parallel lines in the right side of Figure 3. A maximum in *insertion* was observed near a Firmness of 4.26 and Size of 3.31 g.

When comparing the two groups (Figure 3, left vs right), women in the without-applicator group (right) felt a firmer product of moderate Size would be easiest to insert; women in the with-applicator group (left) preferred smaller microbicide prototypes in terms of *insertion*.

#### Willingness

**Figure 4:**
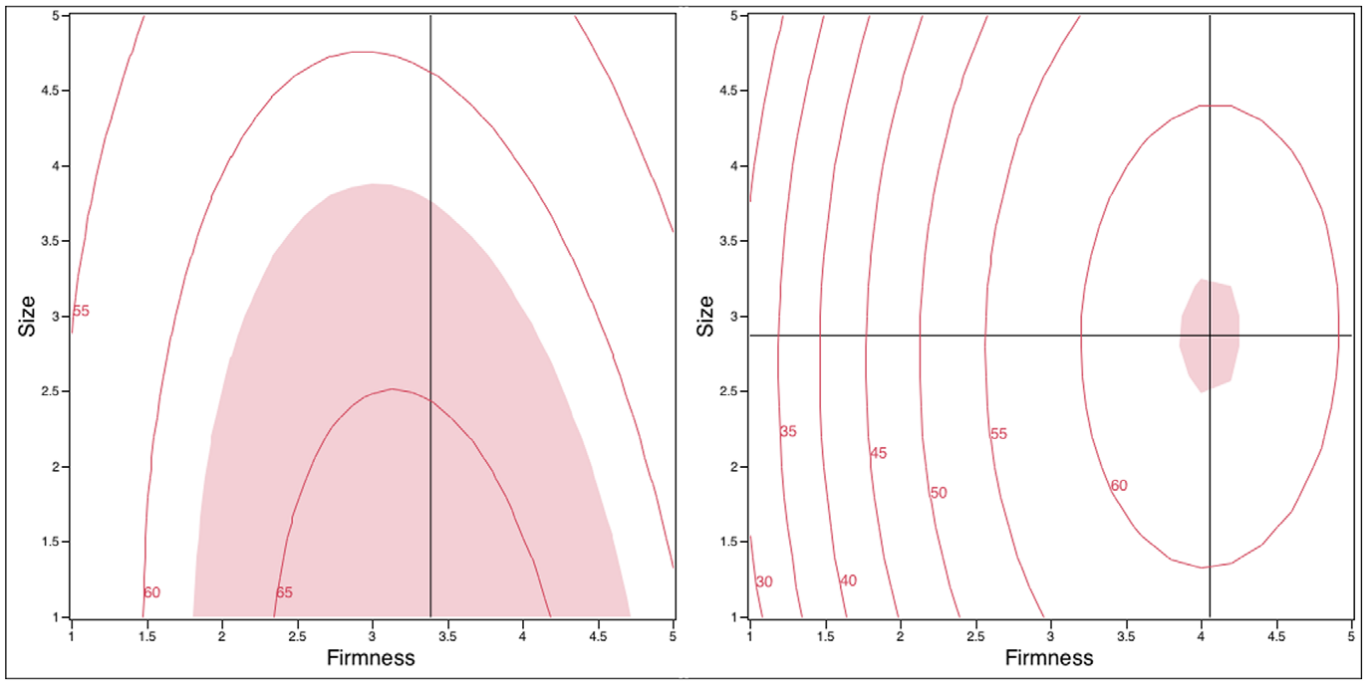
Contour plots of *willingness* (0–100) as a function of design variables (Firmness and Size) in the with-Applicator (left) and without-Applicator groups (right). The cross hairs indicate the location of the maximum value; the optimal size for the with-applicator group was below 1 (outside the design space) and is not shown. The shaded region is the same value (62.3) for both plots.

Effects of Firmness and Size on *willingness* were modeled and are displayed as contour plots (Figure 4).

In the with-applicator group, the RSM model explained 99.4% variation of mean *willingness* scores and the model was significant (F_5,3_ = 169.94, p = 0.0007). The interaction of Firmness by Size was significant (F_1,3_ = 28.52, p = 0.0128). This indicates the influence of Firmness on mean *willingness* scores differs as a function of Size. Both linear (F_1,3_ = 13.66, p = 0.0344) and quadratic terms (F_1,3_ = 466.23, p = 0.0002) of Firmness showed a significant effect. No quadratic effect was found for Size (F_1,3_ = 9.18, p = 0.0563) but its linear terms showed a significant effect (F_1,3_ = 262.01, p = 0.0005). In the contour plot (Figure 4, left), *willingness* increased with increasing Firmness until it reached a level about 3.3; after this maximum, *willingness* declined as Firmness increased. The largest effect of Size was seen near optimum Firmness (right side of left plot in Figure 3): as Size increased, *willingness* declined. An maximal *willingness* value was observed near Firmness = 3.39; again, the optimal size fell outside the design space (below 1 g).

For the without-applicator group (Figure 4, right), the RSM model was significant (F_5,3_ = 23.24, p = 0.0132), explaining of 97.4% of the variation in mean *willingness*. The interaction of Firmness by Size (F_1,3_ = 0.09, p = 0.776) was not significant. Regarding Firmness, both the linear (F_1,3_ = 88.37, p = 0.0026) and quadratic (F_1,3_ = 25.96, p = 0.0146) terms were significant. Conversely, for Size, neither the linear (F_1,3_ = 0.31, p = 0.6192) nor quadratic (F_1,3_ = 2.54, p = 0.2091) terms were significant. Below Firmness 3, Size had little influence on *willingness*. Near optimal Firmness (around 4), Size had a small influence on *willingness*, as the extremely large and small samples were liked less. An optimal *willingness* value was observed near Firmness = 4.06 and Size = 2.87 g, although a wide range of sizes were acceptable at this Firmness level, as Size did not show a strong effect.

When comparing users across groups (Figure 4, left vs right), users in the without-applicator group preferred a slightly firmer product that was also larger. This is consistent with our focus group results (reported elsewhere), as women desire a firmer, larger product that is easier to hold if they are going to use their fingers to insert the microbicide.

### Correlations between Effectiveness, Insertion and Willingness

**Figure 5:**
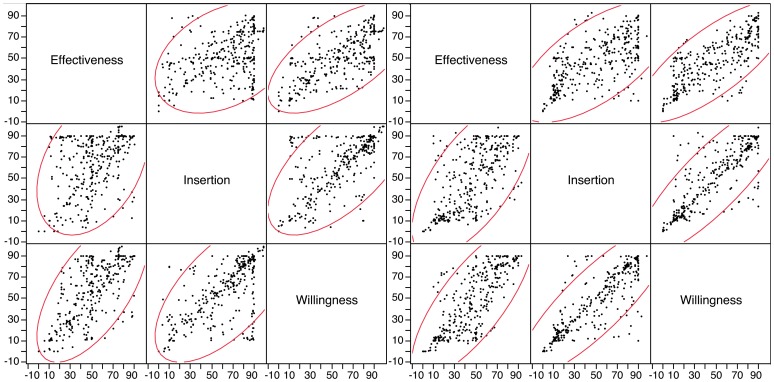
Correlations between *effectiveness*, *insertion* and *willingness* in the with-applicator (left) and without-applicator groups (right).

Within an Applicator condition, *effectiveness*, *insertion* and *willingness* scores were highly correlated (p-values<0.0001) (Figure 5), suggesting they contain largely redundant information. However, the strength of these correlations also differed across the two Applicator conditions. For example, the correlation between *effectiveness* and *insertion* was much lower in the with-applicator group than the without-applicator group (r = 0.37 versus r  = 0.65). Likewise, the correlation between *insertion* and *willingness* was lower in the with-applicator group than the without-applicator group (r  = 0.64 versus r  = 0.84). Finally, *effectiveness* and *willingness* were less correlated in the with-applicator group than those in the without-applicator group (r = 0.64 versus r  = 0.77). Collectively, these results reinforce the view that the presence of an applicator plays a critical role in how Size and Firmness influence user perceptions of *effectiveness, insertion* and *willingness*.

## Discussion

The most significant feature of a microbicide is that use can be initiated by women prior to sex [7], [30]. Microbicides can take many forms (capsules, creams, gels, etc) that range from solid (e.g., capsules) to liquid (creams, ‘gels’). From a sensory perspective, these physical properties can be perceived as hardness, thickness, slipperiness, sticky, watery, runny, etc (e.g., [26]). These attributes may have critical influence on user acceptability of microbicides, and they present a broad, multidimensional design space for formulating a microbicide product. In previous qualitative research by our team [29], users showed a strong preferences for different microbicide prototypes based on size and shape. The influence these design variables and their interactions have on user acceptability have not been well investigated in the field of microbicide development.

Here, we applied standard user-oriented product optimization techniques to ovoid semisoft microbicides in a convenience sample of sexually active women. We found that both Size and Firmness influenced willingness-to-try (*willingness*), imagined ease-of-insertion (*insertion*), and perceived effectiveness (*effectiveness*). Overall, Firmness of microbicide prototypes was more influential on these response variables when compared to Size. We also found that the influence of Size and Firmness differed dramatically as a function of the presence or absence of an applicator. This suggests an applicator plays a critical role in user perceptions of the effect of microbicide firmness and size on product functionality.

### Factors that Influence Microbicide Acceptability

User acceptability of a microbicide is a complex subject [31] involving numerous factors that may potentially influence user behavior [32]. Acceptability is determined by factors related to both the end-user, as well as the specific product characteristics [33]. Regarding the user, factors may include social status, age, cultural norms, and sexual practices and behaviors. Regarding the product, factors related to functionality and sensory properties (e.g., texture, size and shape) are particularly important. These aspects are vital for user acceptance and adherence. Young women in the United States had negative reactions toward ‘gel’ microbicides, as leakage reminded them of uncomfortable sensations present during menstruation, while young women in Puerto Rico felt a microbicide gel was helpful for douching [34]. In one Phase I trial, the microbicide had to be reformulated after users complained about messiness due to too much volume (5 ml) [35]. In the BufferGel study, the microbicide was perceived as “wet” or “sticky” by some women [36]. In an effectiveness study, women preferred a thick or slipperier microbicide instead of a watery one [37]. Lubrication was regarded as an important character for a majority of women and men; however, users disliked a microbicide that was too messy or too wet [38]. In a study of Carraguard in Thailand, most women (95%) believed their partners would be happy to use a microbicide [39].

### Influence of Size and Firmness on *Willingness*, *Insertion,* and *Effectiveness* Depends on Presence of an Applicator

Regarding *willingness*, women in the without applicator group preferred a firmer product that was larger than women in the with-applicator group. This is consistent with qualitative data collected in focus groups [29], where women desired a firmer, larger product that was easier to hold if they were going to use their fingers to insert the product. In the with-applicator group, Size mattered more when the Firmness was close to an optimal level, although, Size was largely irrelevant at extreme Firmness levels. Also, we note that the optimal Size for the with-applicator group was near the edge of our design space, suggesting these results should be interpreted cautiously.

Regarding *insertion*, results across applicator groups were similar to the *willingness* results above: the with-applicator group thought a smaller product would be easier to insert, while the without-applicator group thought a moderately sized microbicide would be easiest to insert. Both groups felt softer microbicides would be difficult to insert. Again, the optimal product for the with-applicator group was near the edge of our design space, suggesting these results should be interpreted cautiously. Additional work with a broader design space would be needed to further optimize applicator based delivery systems.

Regarding *effectiveness*, Firmness influenced *effectiveness* in the without-applicator group, but not the with-applicator group. Presumably, this is because ability to insert a product is necessary (but not sufficient) for it to be effective. That is, *effectiveness* and *insertion* are decoupled in the with-applicator condition (r  = 0.37) whereas in the without-applicator condition, the relationship between *effectiveness* and *insertion* is much higher (r  = 0.65). This is consistent with the premise that you have to be able to insert a microbicide before it can work. Size was not a significant predictor in response surface models of *effectiveness* in either applicator condition. However, the ANOVA results paint a slightly different picture. Size predicted *effectiveness*, but in a non-linear fashion: the smallest Size (1 g) had lower effectiveness scores than the 3 g and 5 g prototypes.

It is also worth noting that the range of *willingness*, *insertion* and *effectiveness* scores are much larger in the without-applicator group than in the with-applicator group. This can be seen in the range of values in the contour plots. For example, in Figure 4, the largest contour is 25% higher than the lowest contour for the with-applicator group, whereas for the without-applicator group, the largest contour is 119% higher than the lowest contour. This difference in range across the two conditions is wholly consistent with a large influence for the presence or absence of an applicator on user acceptability.

### Partial Redundancy of *Willingness*, *Insertion*, and *Effectiveness* Ratings

*Effectiveness*, *insertion* and *willingness* scores were correlated within an applicator condition, suggesting some degree of redundancy among three outcome variables. However, as noted above, the correlations differed across two applicator conditions: generally, the correlations in the without-applicator group were stronger than those in the with-applicator group. This implies presence of an applicator moderates the perceived relationships between the outcome measures. These data support that a one-size-fits-all approach will not work, and microbicides need to be optimized separately depending on whether or not an applicator will be provided.

### Limitations and Conclusions

The current proof of concept study demonstrates that Firmness and Size of microbicide gels influence *effectiveness, insertion* and *willingness*, and the relative influence of each is contingent on whether or not the product is intended to be used with an applicator. However, the present cohort was predominantly middle aged, sexually active, white women in Pennsylvania. This convenience sample was recruited from a rural university campus, and most were married, so these individuals are presumably at low risk for HIV and other STIs. Therefore, the optimum values shown here may not generalize to other groups or populations (e.g., commercial sex workers, adolescents, cultures with different vaginal practices). Also, we did not assess how prior experiences with other vaginal products may have influenced present findings. Firmness and opacity were confounded in the present study, as different levels of carrageenan were required to achieve different levels of Firmness; how color and opacity may influence our outcome measures are unknown. Nonetheless, present data indicate that sexually active women care about product factors such as size and firmness. Future work should include women from more diverse populations, especially populations at high risk for STIs including HIV.

User acceptability of microbicides is a complex subject that is multifactorial and multidimensional. Current variables (shape, firmness and size) were suggested by our qualitative research, and systematically manipulated by our team through product formulation and design. However, design of an optimal microbicide gel need not be limited to these attributes. Additionally, this was a preclinical study and current evaluation was conducted among women who manipulated prototypes *in mano* (in the hand, rather than using them vaginally). Whether or not this generalizes to acceptability in the vagina is unknown. Nonetheless, any vaginal product must be ‘good to think, before it is good to use’. That is, a product with low willingness to try *ex vivo* is highly unlikely to be successful *in vivo*. Finally, to truly understand use acceptability, other external factors should be included in future research, such as culture and ethnicity, race, partnership types and partner opinion, as partners also play an important role in microbicide acceptability [38]. Moreover, assessment of user acceptability can not only guide product design but also help to establish strategies for effective product launches once microbicides are ready for market.

## Supporting Information

Video S1 **All participants watched the concept orientation video at individual viewing stations (laptops with headphones) in our facility waiting room prior to providing informed consent.** The video explains the overall goals of the project, followed by a demonstration of the standardized in mano protocol participants should use to manipulate the prototypes in the test booths.
(M4V)
